# Feature Papers in Food Chemistry—2nd Edition

**DOI:** 10.3390/molecules28227518

**Published:** 2023-11-10

**Authors:** Mirella Nardini

**Affiliations:** CREA, Research Centre for Food and Nutrition, Via Ardeatina 546, 00178 Rome, Italy; mirella.nardini@crea.gov.it

This Special Issue entitled “Feature Papers in Food Chemistry—2nd edition” is a collection of relevant, open access, high-quality papers (original research articles or comprehensive review papers). This Special Issue presents new knowledge or new cutting-edge developments in food chemistry. Particularly, this Special Issue collects manuscripts on food composition, with a special emphasis on chemical characterization, bioactive compounds, contaminants, and analytical aspects (contributions 1–8). 

Oxidative stress is involved in the pathology of many human pathological conditions, such as atherosclerosis, diabetes, ageing, cancer, neurodegenerative, and cardiovascular diseases. The bioactive compounds present in human diets may offer protection against oxidative stress and counteract the onset and development of pathological conditions. Among the dietary bioactive compounds, polyphenols and carotenoids are, by far, the most abundant in human diets, being present in high quantities in fruits and vegetables. Evidence from epidemiological studies suggest that the long-term consumption of polyphenol-rich foods afford the consumer protection against the development of cardiovascular and degenerative diseases, cancer, and diabetes. For individuals that regularly consume wine, beer, coffee, and tea, these beverages represent the main sources of dietary polyphenols. Dietary polyphenol compounds are quickly absorbed and metabolized in humans. 

This Special Issue collects manuscripts on the polyphenolic composition of wine and beer, with a particular interest on the effect of technological processes on the nutritional quality and polyphenols composition of these beverages. As a novelty, the effect of adjuncts, such as fruits, spices, herbs, and natural products, during wine and beer production on the nutritional values of these beverages is presented. In fact, the addition of adjuncts during wine and beer making is preferred by consumers in response to demands for healthy food and new gustatory and olfactory stimuli, improving wine and beer quality.

As a further novelty, the identification and quantitation of single phenolic molecules in beer brewed with or without adjuncts are reviewed and presented for all the classes of phenolic molecules under study, namely phenolic acids, flavonoids, prenylflavonoids, lignans, alkylresorcinols, stilbenes, and phenolic alcohols. 

The roles of variety, cultivation, growing conditions, and technological processes on the nutritional quality of foods and bioactive compounds were also considered, in addition to the role of contaminants, such as aristolochic acid, a nitrophenanthrene carboxylic acid with carcinogenic, mutagenic, and nephrotoxic effects, released by *Aristolochia* plants in soil, causing contamination. 

The relevance of technological processes in improving the nutritional value of food during production, storage, and packaging and in reducing the use of antibiotics was also considered together with their application in the fields of food, flavour and fragrance, pharmaceuticals, chemicals, coatings, and dyes. 

Finally, analytical aspects dealing with the characterization of bioactive molecules were taken into consideration, such as fluorescence and FTIR and NMR spectroscopies, and used as tools for wine authentication and identification.

In spite of the many studies describing the composition of bioactive compounds and the nutritional value of food, the bioavailability, metabolism, and mechanisms of action of bioactive compounds still need to be studied in the depth.

